# Prevalence of skin cancers in kidney transplant recipients - one-center experience

**DOI:** 10.3389/fonc.2025.1636411

**Published:** 2025-10-10

**Authors:** Fabian Kosko, Alicja Dębska-Ślizień, Beata Imko-Walczuk, Maria Luiza Piesiaków, Bogdan Biedunkiewicz, Barbara Bułło-Piontecka, Beata Bzoma, Andrzej Chamienia, Justyna Gołębiewska, Joanna Konopa, Ewa Król, Tomasz Liberek, Monika Lichodziejewska-Niemierko, Sławomir Lizakowski, Przemysław Rutkowski, Agnieszka Tarasewicz, Marcin Wczysla, Zbigniew Heleniak

**Affiliations:** ^1^ Department of Nephrology, Transplantology and Internal Medicine, Medical University of Gdańsk, Gdańsk, Poland; ^2^ Copernicus Hospital, Independent Public Healthcare, Dermatology and Venereology Clinic, Gdańsk, Poland; ^3^ Private Practice in Dermatology and Esthetic Medicine, Gdynia, Poland; ^4^ Palliative Medicine Facility, Medical University of Gdańsk, Gdańsk, Poland; ^5^ Department of Internal Medicine and Pediatric Nursing, Faculty of Health Sciences, Medical University of Gdańsk, Gdańsk, Poland; ^6^ Faculty of Medicine, Medical University of Gdańsk, Gdańsk, Poland

**Keywords:** skin neoplasms, kidney transplantation, squamous cell, basal cell, carcinoma, melanoma

## Abstract

**Introduction:**

Non-melanoma skin cancers (NMSCs) are the most common neoplasms that occur in solid organ transplant recipients (SOTRs). Squamous cell carcinoma (SCC) and basal cell carcinoma (BCC) make up over 90% of skin cancers in SOTRs. Key risk factors include age at transplantation, skin type, immunosuppression, as well as sun exposure (ultraviolet radiation) and viral infections, contributing significantly to tumor development. This study aimed to estimate the incidence and risk of NMSCs in kidney transplant recipients (KTRs) in Poland and to provide new clinical data on patients developing skin cancers in this population.

**Methods:**

This study included 105 KTRs, out of approximately 1,500, who were under the care of the Transplant Outpatient Clinic at the University Hospital in Gdansk between 1980 and 2022 and were diagnosed with NMSC.

**Results:**

A total of 250 cutaneous malignancies were diagnosed in 105 KTRs. BCC (58.8%) and SCC (37.6%) were the most common histological types, and the SCC: BCC ratio was approximately 2:3. Other skin neoplasms, including malignant melanoma (2%) or hidradenocarcinoma, were significantly less frequent. The mean age of KTRs at the time of skin cancer diagnosis was 59.6 years, with a mean time from transplantation to cancer diagnosis of 103.2 months. Most skin cancers were diagnosed 5–10 years post-transplantation and were located on the.24%). The immunosuppressive therapy protocol did not significantly affect the risk of developing skin cancer. The only significant factor associated with an increased risk of skin cancer was patient age. One patient died due to metastatic SCC.

**Conclusion:**

NMSCs account for 90% of skin cancers in KTRs; they have a high recurrence rate and are most often found on the face of older patients towards the end of the first decade after transplantation. Our study confirms that the risk of further skin neoplasm is high and that SCC can be a cause of death. Early detection not only improves prognosis but also minimizes the extent of surgical interventions, which is particularly crucial for lesions in visible areas.

## Introduction

Non-melanoma skin cancers (NMSCs) especially squamous cell carcinoma (SCC) and basal cell carcinoma (BCC) are the most common neoplasms that occur in solid organ transplant recipients (SOTRs) accounting for almost 40 percent of all malignancies in such population and develop in more than 50 percent of White organ transplant recipients and approximately in 6 percent of non-White patients. Altogether, SCC and BCC constitute more than 90 per cent of cutaneous malignancies in SOTRs (1, 2). Although the incidence of both tumor types is markedly increased in SOTRs, the rate of SCC is disproportionately higher. Compared to the general population, the incidence of BCC is increased 10-fold to 16-fold, while SCC occurs at a frequency between 65 and 250 times higher (3). This results in the inversion of the SCC/BCC ratio of 1:4 in the general population to a ratio of at least 4:1 in SOTRs. This reversal becomes even more pronounced with decreasing latitude (i.e., in sunnier climates) and length of time after transplant (3). In SOTRs, as in the general population, SCC presents as a red scaly plaque, typically in sun-exposed areas, and lesions are typically solitary. When it comes to BCC, it usually appears as flesh- or pink-colored, pearly papules with overlying ulceration or telangiectatic vessels, mostly arising on sun-damaged skin (4, 5).

The risk of developing cutaneous malignancies depends on the patient’s age at transplant, skin type, sun exposure, type of immunosuppression and oncogenic viral infections. The mean interval between transplantation and diagnosis of NMSCs varies with patient age at transplantation: 8 years for patients transplanted around 40 years of age, but approximately 3 years for those transplanted after 60 years of age (3). Another risk factor is ultraviolet radiation (UVR) exposure, the mechanism is complex and involves a combination of UV-induced immune suppression, generation of reactive oxygen species and DNA damage (6). One should also mention oncogenic viruses, such as human papillomavirus (HPV), which seem to be involved as a cofactor in the early onset of cutaneous SCC (7).

A particularly critical factor is immunosuppressive (IS) therapy, which nowadays typically consists of a three-drug regimen: glucocorticoids (GKS), calcineurin inhibitors (CNIs), and antiproliferative drugs (APDs). The most commonly used CNIs include cyclosporine A (CSA) and tacrolimus (TAC), while APDs are represented by azathioprine (AZA) and mycophenolate mofetil or sodium (MMF/MPS). Immunosuppressive therapy, however, acts as a double-edged sword. On one hand, it is indispensable for preventing graft rejection; on the other, it significantly heightens the risk of malignant neoplasms, including skin cancers. The mechanisms underlying this phenomenon are multifactorial. IS drugs impair immune surveillance, which normally plays a vital role in identifying and eliminating premalignant and malignant cells. Moreover, Buell et al. showed that CNIs can directly promote tumor growth by fostering angiogenesis and suppressing deoxyribonucleic acid (DNA) repair mechanisms.

Furthermore, the cumulative exposure to immunosuppressants over time amplifies these effects, making long-term transplant recipients particularly vulnerable (8). Although less data is available on TAC compared to CSA, studies suggest that TAC also promotes oncogenesis (9). Regarding APDs, particularly AZA, evidence indicates that it interferes with the post-replicative DNA mismatch repair system, which may contribute to genomic instability and cancer development (10). In contrast, MMF has been suggested to exert a protective effect against cancer, although its precise role remains inconclusive and requires further investigation (9). Moreover, MMF increases the potency of immunosuppression, which promotes post-transplant viral infections and associated cancers by impairing immune response against viruses and cancer immunoediting (11, 12). Evidence from some studies suggests that mammalian target of rapamycin inhibitors (mTORi) may be associated with a reduced risk of NMSC cancer development (13, 14). Knoll et al., in their huge metanalysis (5876 patients from 21 randomized trials), observed that the time to first NMSC in kidney transplant recipients (KTRs) according to immunosuppressive treatment was significantly longer in patients receiving sirolimus (SRL). However, this individual patient-level meta-analysis of nearly 6000 KTRs found that SRL significantly reduced NMSC, but it increased death. The risk was not observed in the low dose SRL group (below the median sirolimus drug concentration of 10 ng/mL). Therefore, SRL should be used with caution in KTRs, given the excess risk of mortality when applied in high doses (15).

This study aimed to estimate the incidence rate and risk of NMSCs in KTRs under the care of a large transplant unit in the Pomerania region of Poland. One of the goals was also to obtain new clinical data on patients who are developing skin cancers.

## Methods

This study included patients who underwent kidney transplantation (KTx) between 1980 and 2022 and were under the care of the Transplant Outpatient Clinic at the University Hospital in Gdansk, Poland. Patients were enrolled in the study if they had a histopathologically confirmed diagnosis of skin cancer. A total of 105 patients, out of approximately 1,500 (7%), meeting these criteria were included in the analysis. All the cases of skin malignancies, which occurred in the studied group from 1980 to December 2023, were included into the study. To our knowledge, this study appears to be the largest which was conducted in Poland. The study was performed in accordance with ethical guidelines and approved by the Bioethics Committee of the Medical University of Gdansk (MUG) (Approval No KB/446/2023).

### Statistical analysis

The statistical analysis was conducted by the Centre of Biostatistics and Bioinformatics Analysis of the MUG. Binary variables were analyzed using Fisher’s exact test. For categorical variables with more than two levels, Fisher’s exact test with the Freeman-Halton extension was applied. The normality of quantitative variables was assessed using the Shapiro-Wilk test. For normally distributed variables, comparisons were made using the t-test; otherwise, the Mann-Whitney U test was applied. A p-value of < 0.05 was considered statistically significant. No data imputation was performed. In survival analyses, the Schoenfeld test was used to evaluate the proportional hazards assumption, and no variables were found to violate this assumption. Hazard ratios (HR) were determined using univariate and multivariate Cox regression. Bootstrapping was employed to estimate confidence intervals for the median progression-free survival.

## Results

### Characteristics of KTRs with cutaneous malignancies

A cohort of 105 KTRs, all of whom were Caucasian, was included in the study. The vast majority of the patients were male (n = 81; 77.14%). The mean age at the KTx was 50.9 ± 12.2 years (49.5 ± 12.9 for women and 51.3 ± 12.1 for men), and the mean follow-up time calculated from the date of KTx was 181.6 months. The most common causes of end-stage kidney disease among the patients were chronic glomerulonephritis (GN) - 41 patients (39.05%), autosomal dominant polycystic kidney disease (ADPKD) - 18 patients (17.14%), interstitial nephritis (IN) – 10 patients (9.52%), hypertensive nephropathy (HTN) - 6 patients (5,71%), diabetic kidney disease (DKD) - 4 patients (3.81%). In 26 patients (24.76%), it was caused by different diseases (e.g. gout, drug-induced nephropathy) or the cause of chronic renal failure had not been established. In the studied group, KTRs underwent dialysis on average for 24 months (range of 0-120.0) before the renal transplantation. The most frequent type of renal replacement therapy (RRT) was hemodialysis (HD) - 88 patients (86.27%), peritoneal dialysis (PD) - 8 patients (7.84%), and 6 patients (5.88%) underwent preemptive kidney transplantation (PKT). Detailed demographic and clinical characteristics of the study group are presented in **Table 1**.

**Table 1:** Detailed demographic and clinical characteristics of the study group.

	All	Males	Females	p-value
N	N = 105	N = 81	N = 24	
Age of recipient	50.867 (12.248)	51.284 (12.092)	49.458 (12.928)	0.5415^b^
Cause of ESKD	N = 105	N = 81	N = 24	
ADPKD	18 [17.14%]	12/81 [14.81%]	6/24 [25%]	0.0876^a^
DM	4 [3.81%]	2/81 [2.47%]	2/24 [8.33%]	
GN	41 [39.05%]	30/81 [37.04%]	11/24 [45.83%]	
HA	6 [5.71%]	6/81 [7.41%]	0/24 [0%]	
Interstitial nephritis	10 [9.52%]	7/81 [8.64%]	3/24 [12.5%]	
Other or unknown etiology	26 [24.76%]	24/81 [29.63%]	2/24 [8.33%]	
Time of RRT	N = 95	N = 72	N = 23	
	24 (0-120)	24 (0-120)	24 (0-108)	0.855^c^
Type of RRT	N = 102	N = 78	N = 24	
HD	88 [86.27%]	67/78 [85.9%]	21/24 [87.5%]	1^a^
PD	8 [7.84%]	6/78 [7.69%]	2/24 [8.33%]	
Preemptive	6 [5.88%]	5/78 [6.41%]	1/24 [4.17%]	
Number of KTx	N = 105	N = 81	N = 24	
1	87 [82.86%]	67/81 [82.72%]	20/24 [83.33%]	0.822^a^
2	14 [13.33%]	10/81 [12.35%]	4/24 [16.67%]	
3	3 [2.86%]	3/81 [3.7%]	0/24 [0%]	
No data	1 [0.95%]	1/81 [1.23%]	0/24 [0%]	
Histology type	N = 105	N = 81	N = 24	
BCC	147 (58.8%)			
SCC	94 (37.6%)			
MM	4 (1.6%)			
Others	4 (1.6%)			
Scheme	N = 105	N = 81	N = 24	
CSA + AZA	3 [2.86%]	1/81 [1.23%]	2/24 [8.33%]	0.0263 *^a^
GKS + AZA	1 [0.95%]	0/81 [0%]	1/24 [4.17%]	
GKS + CSA	3 [2.86%]	1/81 [1.23%]	2/24 [8.33%]	
GKS + CSA + AZA	11 [10.48%]	7/81 [8.64%]	4/24 [16.67%]	
GKS + CSA + MMF	40 [38.1%]	31/81 [38.27%]	9/24 [37.5%]	
GKS + TAC	4 [3.81%]	4/81 [4.94%]	0/24 [0%]	
GKS + TAC + AZA	3 [2.86%]	3/81 [3.7%]	0/24 [0%]	
GKS + TAC + MMF	31 [29.52%]	28/81 [34.57%]	3/24 [12.5%]	
Other	9 [8.57%]	6/81 [7.41%]	3/24 [12.5%]	
GKS	N = 105	N = 81	N = 24	
no	7 [6.67%]	3/81 [3.7%]	4/24 [16.67%]	0.0464 *^a^
yes	98 [93.33%]	78/81 [96.3%]	20/24 [83.33%]	
CSA	N = 104	N = 80	N = 24	
no	47 [45.19%]	41/80 [51.25%]	6/24 [25%]	0.0344 *^a^
yes	57 [54.81%]	39/80 [48.75%]	18/24 [75%]	
TAC	N = 105	N = 81	N = 24	
no	63 [60%]	43/81 [53.09%]	20/24 [83.33%]	0.0089 **^a^
yes	42 [40%]	38/81 [46.91%]	4/24 [16.67%]	
MMF.	N = 105	N = 81	N = 24	
no	31 [29.52%]	21/81 [25.93%]	10/24 [41.67%]	0.2015^a^
yes	74 [70.48%]	60/81 [74.07%]	14/24 [58.33%]	
AZA	N = 105	N = 81	N = 24	
no	82 [78.1%]	66/81 [81.48%]	16/24 [66.67%]	0.16^a^
yes	23 [21.9%]	15/81 [18.52%]	8/24 [33.33%]	
SRL	N = 105	N = 81	N = 24	
no	101 [96.19%]	78/81 [96.3%]	23/24 [95.83%]	1^a^
yes	4 [3.81%]	3/81 [3.7%]	1/24 [4.17%]	

^a^Fisher test; ^b^T-test; ^c^Mann-Whitney U test; ADPKD, autosomal dominant polycystic kidney disease; AZA, azathioprine; CsA, cyclosporine A; DM, diabetes mellitus; ESRD, end-stage renal disease; GKS, glucocorticosteroids; GN, glomerulonephritis; HD, hemodialysis; HA, Hypertension; IS, immunosupressive; KTx, kidney transplantation; MMF, mycophenolate mofetil or mycophenolate sodium; PD, peritoneal dialysis; RRT, renal replacement therapy; TAC, tacrolimus.

Most patients included in the study underwent only one kidney transplantation procedure (82.86%), while 13.33% and 2.86% received two and three kidney transplants, respectively. There was, however, no statistically significant correlation between the number of skin cancers and the number of kidney transplants (p = 0.1962).

The initial immunosuppressive therapy following the first KTx in our cohort comprised GKS (n=98; 93.33%), CSA (n=57; 54.81%), TAC (n=42; 40.0%), MMF (n=74; 70.48%), AZA (n=23; 21.9%), and sirolimus (n=4; 3.81%). We did not have access to data regarding prior immunosuppressive therapy administered before kidney transplantation, nor information about the history of NMSCs and other cancers before the transplantation.

### Characteristics of skin cancers observed among KTRs

**Figure 1:**
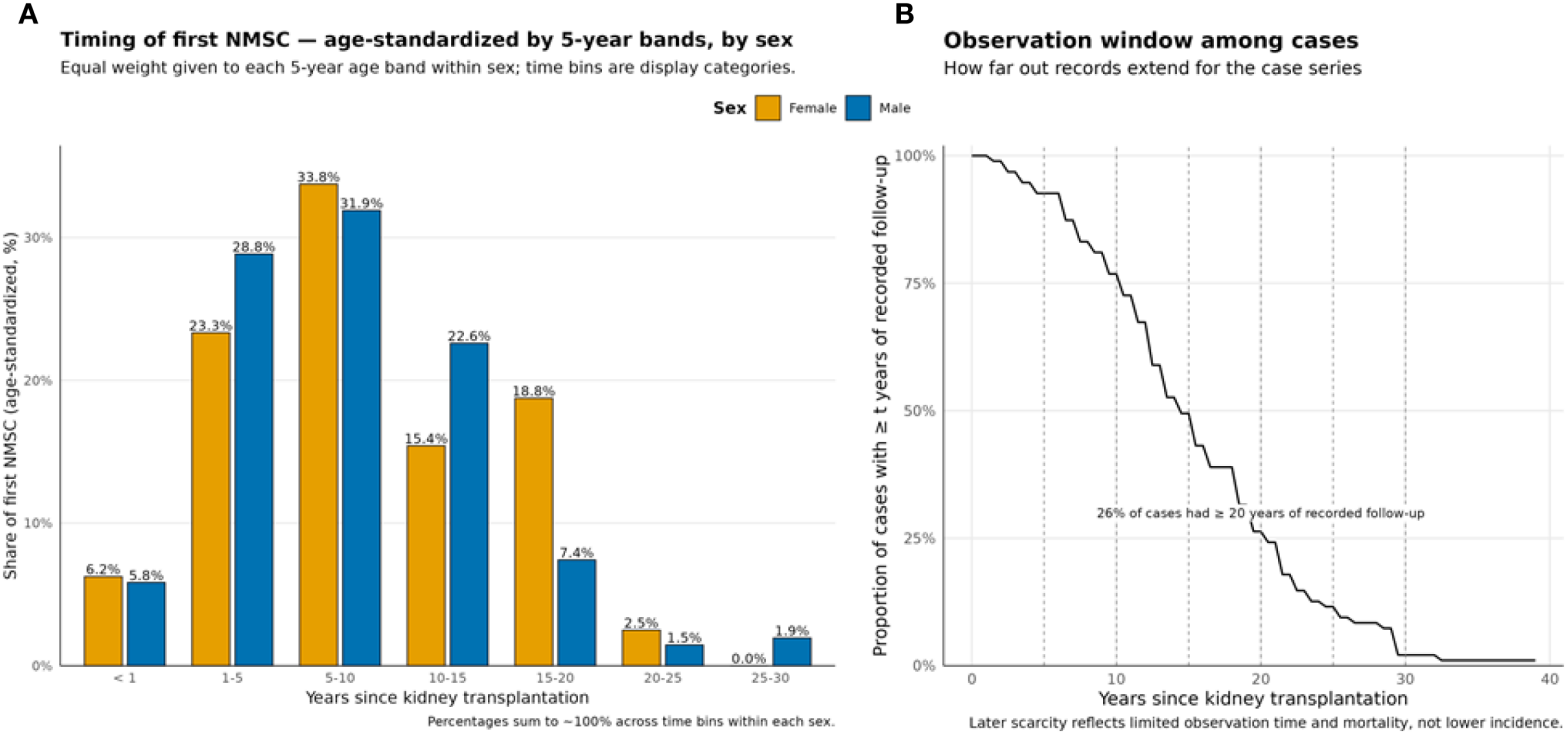
Occurrence of first NMSC post-kidney transplantation. **(A)** Age standardized shareby time bin and sex (equal weights per 5-year age band). **(B)** Proportion of cases by follow-up time (t) duration; dashed lines mark bin boundaries.

A total of 250 cutaneous malignancies were diagnosed in 105 KTRs included in the study. The mean age of the recipient at the time of cancer development was 59.6 years, with a mean time from transplantation to cancer diagnosis of 103.2 months. The most first cancers were diagnosed within the time frame of 5–10 years after KTx (**Figure 1A**), with a higher counts observed in male patients compared to females across all time periods. A notable decline in the incidence was observed after 15 years post-transplantation; however, this pattern reflects the limited observation window in the case series, with only 26% of cases having ≥20 years of recorded follow-up (**Figure 1B**). The distribution of first NMSC cases in time intervals, divided by sex, and the supporting observation window are shown in **Figure 1A**.

The most common skin malignancies observed were BCC (58.8%) and SCC (37.6%); other skin neoplasms, including malignant melanoma (2%) or hidradenocarcinoma, were significantly less frequent. This distribution highlights the predominance of NMSCs in the studied population.

A total of 47 patients (44.8%) developed more than one skin cancer after KTx during the observation period. The mean time from the diagnosis of the first cancer to the occurrence of another one was 2 years (range: 0–10 years), and in 59.6% of cases, the histological type remained the same (42.6% for BCC and 17.2% for SCC). The highest recorded number of skin cancers in a single patient was 16 over a period of 8 years (2008–2016). Among the 33 patients in whom SCC was diagnosed, high-risk features according to the recent National Comprehensive Cancer Network (NCCN) guidelines were present in the vast majority of these cases (N = 28; 84.9%), where the tumor was located on the face, the recurrence incidence was 23.3% and immunosuppressive therapy was present all patients (16, 17).

Cutaneous malignancies were most commonly located on the face (n=79; 75.24%), followed by the limbs (with the upper limbs being more frequently affected than the lower limbs) and the back. Overall, the first skin cancer was located in the head and neck (H&N) region in 78.09% of cases. The frequency of neoplasm localizations is presented in **Table 2**. The site of skin cancer varied significantly by sex (p=0.0103), with males more frequently experiencing facial skin cancer (79.01%) compared to females (62.5%). Moreover, there were no correlation between the number of KTx and number of skin cancers in the follow-up period of time.

**Table 2:** Characteristics of first skin cancers in studied group of KTRs.

First cancer histological type	N = 105	N = 81	N = 24	p-value
BCC	69 [65.71%]	54/81 [66.67%]	15/24 [62.5%]	0.7453^a^
SCC	33 [31.43%]	25/81 [30.86%]	8/24 [33.33%]	
MM + others	3 [2.86%]	2/81 [2.47%]	1/24 [4.17%]	
Localization of first cancer	N = 105	N = 81	N = 24	
Abdomen	1 [0.95%]	0/81 [0%]	1/24 [4.17%]	0.0103 *^a^
Back	3 [2.86%]	3/81 [3.7%]	0/24 [0%]	
Chest	2 [1.9%]	0/81 [0%]	2/24 [8.33%]	
Face	79 [75.24%]	64/81 [79.01%]	15/24 [62.5%]	
Lower Limb	4 [3.81%]	2/81 [2.47%]	2/24 [8.33%]	
Nape of Neck	2 [1.9%]	0/81 [0%]	2/24 [8.33%]	
Scalp	1 [0.95%]	1/81 [1.23%]	0/24 [0%]	
Unknown	6 [5.71%]	5/81 [6.17%]	1/24 [4.17%]	
Upper Limb	7 [6.67%]	6/81 [7.41%]	1/24 [4.17%]	

^a^Fisher test; BCC, basocellular carcinoma; SCC, squamous cell carcinoma; MM, malignant melanoma.

### Patient’s survival

In the analyzed cohort of 105 KTRs, nearly one-fifth (21%) of patients died during the follow-up period. However, skin cancer per se (SCC) accounted for only a single death fortunately, the cause of death for the vast majority of patients remains unknown, which may limit the reliability of this data. Among the known causes, infections were the most common, likely secondary to immune system impairment caused by post-transplant immunosuppression. **Table 3** presents survival outcomes both from the time of cancer diagnosis and since kidney transplantation.

**Table 3:** Time from KTx to cancer diagnosis, follow-up time from cancer diagnosis and follow-up from KTx.

Cancer type	N	Time from KTx to cancer diagnosis median (IQR) [years]	Follow-up from cancer diagnosis median (95% CI) [years]	Follow-up from KTx median (95% CI) [years]
BCC	69	7 (6-9)	5 (3.5-7)	13 (6-9)
MM + other	3	3 (1-18)	12 (11-18)	21 (1-18)
SCC	33	7 (7-10)	8 (2-9)	15 (7-10)
All	105	7 (6-9)	6 (4-8)	14 (6-9)

BCC, basocellular carcinoma; SCC, squamous cell carcinoma; MM, malignant melanoma.

### The relationship between immunosuppressive protocol and cancer

Multivariate Cox proportional hazards analysis confirmed that neither the choice of CNI nor APD significantly influenced the risk of developing skin cancer. CSA served as the reference for CNI, while AZA was the reference for APD. For CNIs, the HR for TAC, compared to CSA, was 0.83 (95% CI: 0.55–1.26; p = 0.384), while for APDs, the HR for MMF, compared to AZA, was 0.85 (95% CI: 0.5–1.46; p = 0.5541). Furthermore, analysis of different IS regimens yielded no statistically significant differences, with HRs close to 1 and p-values >0.2 for all comparisons (**Table 4**).

**Table 4:** KTRs skin cancer free survival.

Variable	Univariate HR (95% CI)	Univariate p	Multivariable HR (95% CI)	Multivariable p
Antiproliferative drug				
Azathioprine	Baseline	–	Baseline	–
MMF	1.29 (0.76-2.17)	0.3428	0.85 (0.5-1.46)	0.5541
None	1.28 (0.53-3.08)	0.5805	0.7 (0.28-1.71)	0.4314
CNI				
CsA	Baseline	–	Baseline	–
Tacrolimus	0.89 (0.59-1.35)	0.5809	0.83 (0.55-1.26)	0.384
None	0.27 (0.04-1.97)	0.1952	0.59 (0.07-4.64)	0.6126
Scheme				
GKS + CSA + MMF	Baseline	–	Baseline	–
GKS + TAC + MMF	0.8 (0.5-1.29)	0.3696	0.86 (0.53-1.38)	0.5275
GKS + CSA + AZA	0.82 (0.42-1.6)	0.5518	0.97 (0.49-1.92)	0.9332
Other	0.97 (0.58-1.63)	0.9166	1.39 (0.82-2.35)	0.2233

AZA, azathiopryne; CSA, cyclosporine A; MMF, mycophenolate mofetil or mycophenolate sodium; TAC, tacrolimus; GKS, glucocorticoids.

After the diagnosis of the first cutaneous malignancy, the IS regimen was changed in a total of 11 patients, mostly by switching from APDs to everolimus (EVE). However, despite the switch, 7 patients developed additional skin cancers (63.6% vs. 85.1% in KTRs without IS regimen conversion). Applied doses of EVE were low (drug concentration below 5 ng/ml), and side effects were not observed.

### The factor significantly associated with an increased risk of skin cancer

The only factor significantly associated with an increased risk of skin cancer was patient age. All multivariable p-values exceeded the significance threshold of 0.05 (**Table 4**); thus, after adjusting for age, the effect of immunosuppressive medications was no longer significant. Consequently, age emerged as the only statistically significant factor influencing skin cancer risk in the adjusted multivariate analysis.

### The relationship between immunosuppressive protocol and patients’ survival

In this study, we evaluated the impact of immunosuppressive regimens on the development of skin cancer after KTx. Kaplan-Meier survival analysis and HRs were used to assess the cancer-free survival concerning various immunosuppressive protocols.

**Figure 2:**
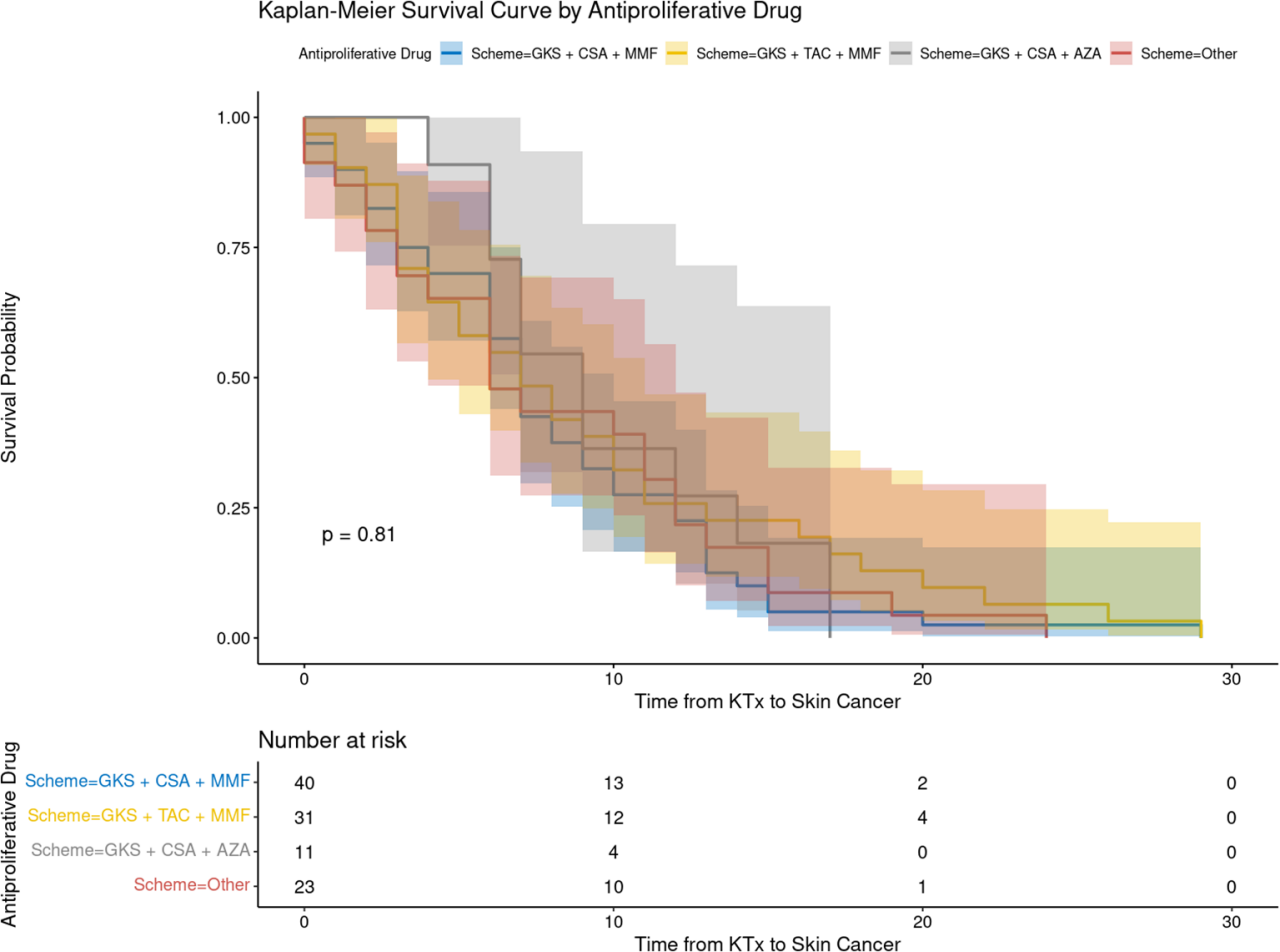
The recipient’s cancer-free survival following kidney transplantation depending on immunosuppressive protocol. AZA, azathiopryne; CsA, cyclosporine A; GKS, glucocorticosteroids; MMF, mycophenolate mofetil or mycophenolate sodium; TAC, tacrolimus.

**Figure 3:**
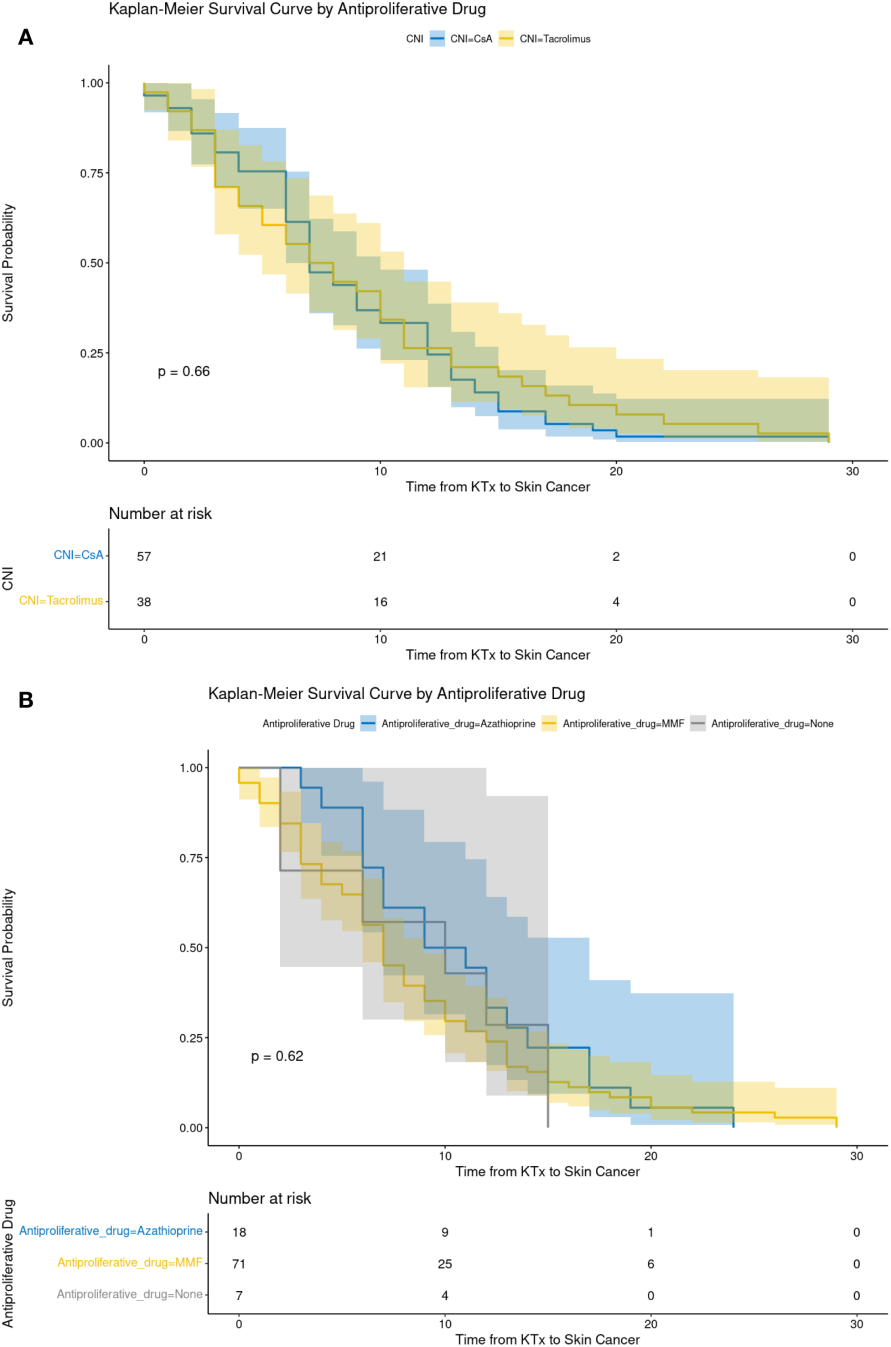
The recipient’s cancer-free survival depending on the choice of calcineurin inhibitor **(A)** and antiproliferative drugs **(B)**. AZA, azathiopryne; CsA, cyclosporine A; MMF, mycophenolate mofetil or mycophenolate sodium; TAC, tacrolimus.

The Kaplan-Meier survival curves (**Figures 2**, **3**) demonstrated no statistically significant differences in skin cancer-free survival between patients receiving different CNIs (TAC vs. CSA, p = 0.66; **Figure 3A**) or APDs (MMF vs. AZA, p = 0.62; **Figure 3B**). Similarly, comparisons between immunosuppressive schemes (the three most common regimens) did not reveal any significant impact on cancer-free survival (p = 0.81; **Figure 2**).

The analysis of immunosuppressive regimens on patient survival following kidney transplantation revealed no statistically significant associations. For APDs, AZA was set as the reference group. MMF demonstrated HR of 1.26 (95% CI: 0.4-3.91) in the univariate analysis and 1.16 (95% CI: 0.36-3.74) in the multivariable analysis, with no statistical significance (p = 0.6924 and p = 0.7994, respectively). Similarly, the absence of an APD was not significant (HR = 1.54; p = 0.7079). For CNI, CSA served as the baseline reference. TAC showed lower HR values (0.34; 95% CI: 0.1-1.17) in the univariate analysis and 0.36 (95% CI: 0.1-1.28) in the multivariable analysis; however, these differences did not reach statistical significance (p = 0.0869 and p = 0.1142, respectively). Regarding treatment regimens, no significant impact on survival was observed among the analyzed groups. Compared to the reference regimen (GKS + CSA + MMF), the HR for GKS + TAC + MMF was 0.8 (95% CI: 0.5-1.29) in the univariate analysis and 0.53 (95% CI: 0.14-1.99) in the multivariable analysis (p > 0.05). Similar results were observed for GKS + CSA + AZA and other therapy combinations (**Table 5**).

**Table 5:** Survival of KTRs depending on IS protocol.

Variable	Univariate HR (95% CI)	Univariate p	Multivariable HR (95% CI)	Multivariable p
Antiproliferative drug				
Azathioprine	Baseline	–	Baseline	–
MMF	1.26 (0.4-3.91)	0.6924	1.16 (0.36-3.74)	0.7994
None	1.22 (0.14-11.02)	0.8596	1.54 (0.16-14.6)	0.7079
CNI				
CsA	Baseline	–	Baseline	–
Tacrolimus	0.34 (0.1-1.17)	0.0869	0.36 (0.1-1.28)	0.1142
None	0 (0-Inf)	0.9983	0 (0-Inf)	0.9981
Scheme				
GKS + CSA + MMF	Baseline	–	Baseline	–
GKS + TAC + MMF	0.8 (0.5-1.29)	0.3696	0.53 (0.14-1.99)	0.3465
GKS + CSA + AZA	0.82 (0.42-1.6)	0.5518	1.13 (0.34-3.78)	0.8394
Other	0.97 (0.58-1.63)	0.9166	0.61 (0.16-2.31)	0.4642

AZA, azathiopryne; CSA, cyclosporine A; MMF, mycophenolate mofetil or mycophenolate sodium; TAC, tacrolimus; GKS, glucocorticoids.

## Discussion

It is well established that patients who have undergone solid organ transplantation, including KTRs, are at an increased risk of developing a wide range of cancers. According to Engels et al., the total standardized incidence ratio (SIR) for cancer development among SOTRs, compared to the general population, was 2.1 (95% CI: 2.06–2.14). However, the SIR value varies between specific cancers. In the case of NMSCs, SIR was significantly higher, at 13.85 (95% CI: 11.92–16.00) (18).

In our study cohort, a total of 250 skin neoplasms were identified among 105 KTRs. The mean age of these individuals at the time of skin cancer diagnosis was 59.6 years, underscoring the prevalence of such malignancies in an older transplant population. The average duration from the time of transplantation to the diagnosis of skin cancer was calculated at 103.2 months, indicating a substantial lag period during which cumulative immunosuppressive exposure likely played a pivotal role. Notably, the majority of skin cancer cases emerged within the 5 to 10 years following transplantation, highlighting this period as a critical window for vigilant dermatological screening. A notable decline in the incidence was observed after 15 years post-transplantation; however, this pattern reflects the limited observation window in the case series, with only 26% of cases having ≥20 years of recorded follow-up due to censoring, mortality, and the study’s historical timeframe (1980-2023).

Multiple factors contribute to the heightened risk of skin cancer in KTRs, with IS therapy being a significant one. As discussed earlier, IS drugs can promote oncogenesis through various mechanisms. Moreover, the cumulative exposure to immunosuppressants over time amplifies these negative effects, making long-term transplant recipients particularly vulnerable (9). However, our study found that no specific IS regimen emerged as a significant predictor of cancer risk. Additionally, the analysis revealed no significant differences in survival outcomes based on the IS protocol used. This lack of association may be due to the low number of skin cancer-related deaths within the studied population. In some patients diagnosed with skin cancer, the IS treatment was modified, predominantly transitioning to one of the mTORi: EVE or SRL. Evidence from several studies suggests that mTOR inhibitors may be associated with a reduced risk of cancer development (13, 14). However, in our study, despite the conversion to EVE, the majority of KTRs (7 out of 11) developed additional malignancies following the switch. One of the potential reasons for this observation could be the carcinogenic effects of prior IS agents, which may have contributed to the accumulation of genetic damage in skin cells over time. Thus, the beneficial effects of EVE may not have manifested. Moreover, the protective effect of mTOR inhibitors appears to be time-dependent (19, 20), however such data are not available in our study population.

Histological analysis in our study population revealed that BCC and SCC were the most frequently occurring neoplasms, together accounting for over 90% of all skin malignancies. In contrast, other skin cancers, such as malignant melanoma (2%), were significantly less common. This finding is consistent with reports in the available literature (3). A notable difference between our findings and the literature lies in the SCC: BCC ratio. While previous studies have reported an approximate 4:1 ratio favoring SCC, in our cohort, BCC was more prevalent. This discrepancy may reflect differences in geographic, genetic and environmental factors influencing skin cancer distribution in transplant populations and also differences in the follow-up time, because in KTRs, the incidence of BCC grows linearly, while the SCC rate grows exponentially (21). In case of geographic differences, the SCC: BCC ratio was reported as 2:1 in Australia (22), whereas in patients from a similar latitude to Poland, like the Czech Republic, the ratio was also 2:3 (23). The relatively short follow-up time in our study is another reason for such SCC: BCC ratio. With a longer follow-up period, the ratio would likely shift, favoring SCC over BCC.

In our population, multivariate models have demonstrated that patient age is a significant factor associated with an increased risk of developing skin cancer. This finding is particularly relevant in the current era, as progressively older patients are being considered eligible for KTx. While this expansion of eligibility criteria is a positive development in terms of providing life-saving treatment to a broader population, it may also contribute to a rise in post-transplant skin cancer cases due to the compounding effects of age-related immunosenescence and cumulative exposure to carcinogenic risk factors.

Other factors, such as oncogenic viruses, are also known to contribute to the pathogenesis of skin cancer in KTRs. However, our study lacks data on viral infections, which limits our ability to evaluate their potential impact on the observed cancer incidence.

As mentioned above, the incidence ratio for NMSC among SOTRs is much higher than in the general population (SIR 13.85) (16). In 2022, a study conducted by the European Academy of Dermatology and Venereology (EADV) demonstrated that the occurrence of skin cancer was reported in 1.71% of the adult European population compared with 7% in our cohort of KTRs (24). The discrepancy between KTRs and the general population may be explained by significant underreporting and undernotification of NMSC cases in the latter group, due to their typically less aggressive nature, limited public awareness, and the lack of mandatory registration for such diagnoses. In the transplant population, however, heightened medical surveillance and an increased risk due to immunosuppression lead to higher detection rates, contributing to the prominence of these cases in this group. Despite data from cancer registries, it is important to highlight that skin cancer is the most common type of malignancy in the Caucasian population (25). Post-transplant surveillance in patients with NMSC using general population guidelines and the higher risk in immunocompromised recipients and particularly those with a history of SCC, surveillance recommendations include a full-body examination by a dermatologist, ideally every 3–12 months, depending on the preexisting tumor, as well as lymph node examination and imaging studies in patients with a more significant history (26).

Due to the high incidence rate of cutaneous cancers among KTRs, it is essential to incorporate a proper education which should begin during the waiting period for KTx and continue throughout the post-transplant phase. The focus should be on primary prevention, including avoiding excessive UV exposure, performing self-skin examinations, and attending regular dermatological check-ups according to guidelines. Additionally, patients should be informed about the importance of secondary prevention following the detection of skin cancer. Regular dermatological screenings are vital, and establishing dedicated dermatology clinics at transplant centers is necessary to ensure early diagnosis and effective treatment. Late diagnosis of invasive SCC presents therapeutic challenges, including the risk of graft dysfunction or even loss due to the need for systemic treatment, as well as cosmetic concerns (27, 28).

To summarize, NMSCs account for 90% of skin cancers in KTRs; they have a high recurrence rate and are most often found on the face of older patients towards the end of the first decade after transplantation. and the BCC prevalence was higher as compare to SCC, what is not in line with other studies with longer transplant population observation time. Therefore, we assume that these proportions may change as the follow-up time of our population increases, because SCC appears later after transplantation than BCC.

Our study confirms that the risk of further skin neoplasm is high and that SCC can be a cause of death. The frequent occurrence of these malignancies emphasizes the critical need for regular dermatological evaluations, particularly focusing on the most common anatomical locations for lesions. Early and systematic screenings can facilitate the detection of precancerous conditions or cancers at their initial stages, leading to improved prognosis. Additionally, early detection reduces the need for extensive surgical procedures, leading to smaller scars and better cosmetic outcomes, which is particularly crucial for lesions in highly visible areas. Regular and frequent dermatological evaluations are crucial in KTRs with NMSC since the following lesions appear early and frequently after primary.

## Limitations

This study was limited by its single-center design, relatively small population, insufficient data to determine whether patients had received immunosuppressive therapy before KTx for underlying conditions such as GN, a lack of information concerning oncogenic viral infections (e.g., HPV), and a lack of information regarding non-cutaneous malignancies before KTx. These limitations may have affected our ability to comprehensively evaluate the impact of pre-transplant immunosuppression on post-transplant outcomes. Moreover, we do not have sufficient data regarding the patients’ history of NMSC prior to kidney transplantation. One potential reason for this could be underreporting or under notification of NMSC cases. In our study, we have also the limited observation window in the case series, with only 26% of cases having ≥20 years of recorded follow-up due to censoring, mortality, and the study’s historical timeframe (1980-2023).

## Data Availability

The original contributions presented in the study are included in the article/supplementary material. Further inquiries can be directed to the corresponding authors.
